# Enhancing emotional experiences to dance through music: the role of valence and arousal in the cross-modal bias

**DOI:** 10.3389/fnhum.2014.00757

**Published:** 2014-10-06

**Authors:** Julia F. Christensen, Sebastian B. Gaigg, Antoni Gomila, Peter Oke, Beatriz Calvo-Merino

**Affiliations:** ^1^Department of Psychology and Human Evolution and Cognition (IFISC-CSIC), University of the Balearic IslandsPalma de Mallorca, Spain; ^2^Department of Psychology, School of Arts and Social Science, City University LondonLondon, UK; ^3^Department of Psychology, Universidad Complutense de MadridMadrid, Spain

**Keywords:** cross-modal, affective body movement, multisensory, neuroentrainment, psychology of emotion, neuroesthetics, arousal, valence

## Abstract

It is well established that emotional responses to stimuli presented to one perceptive modality (e.g., visual) are modulated by the concurrent presentation of affective information to another modality (e.g., auditory)—an effect known as the *cross-modal bias*. However, the affective mechanisms mediating this effect are still not fully understood. It remains unclear what role different dimensions of stimulus valence and arousal play in mediating the effect, and to what extent cross-modal influences impact not only our perception and conscious affective experiences, but also our psychophysiological emotional response. We addressed these issues by measuring participants’ subjective emotion ratings and their Galvanic Skin Responses (GSR) in a cross-modal affect perception paradigm employing videos of ballet dance movements and instrumental classical music as the stimuli. We chose these stimuli to explore the cross-modal bias in a context of stimuli (ballet dance movements) that most participants would have relatively little prior experience with. Results showed (i) that the cross-modal bias was more pronounced for sad than for happy movements, whereas it was equivalent when contrasting high *vs*. low arousal movements; and (ii) that movement valence did not modulate participants’ GSR, while movement arousal did, such that GSR was potentiated in the case of low arousal movements with sad music and when high arousal movements were paired with happy music. Results are discussed in the context of the affective dimension of neuroentrainment and with regards to implications for the art community.

## Introduction

Dance is one of the most expressive types of affective body language. In Béjart’s choreography of *Boléro*, the audience is almost burned by the passion on display (Béjart and Ravel, 1961) and when *Gisèlle* dances her final goodbye to her loved one and returns to the tomb, sobbing is heard from the audience stands (Perrot et al., 1841). Yet, few of us can imagine *dance* without the accompaniment of one of the other grand art forms: music. Music often potentiates the experience of affect expressed in movement; and in theatre and cinema alike, directors use music to great effect in order to regulate the audience’s emotions (Schneider et al., 1994; Baumgartner et al., 2006a; Cohen, 2010).

Psychological research has provided ample evidence to confirm that our affective perception of an emotional stimulus presented to one sensory modality (e.g., visual) is altered by the emotional information presented to another modality (e.g., auditory), even when participants are asked to ignore the latter. This effect is called the *cross-modal bias*, and was first described in the purely perceptual domain, showing that visual perception (e.g., intensity) is enhanced by simultaneous auditory stimuli (Stein et al., 1996; Vroomen and de Gelder, 2000). In the psychology of emotion this effect is typically studied by comparing participants’ perception of affect from a *visual* stimulus under three experimental conditions that include either the presentation of additional *congruent* or *incongruent* affective *auditory* information or no concurrent affective information at all (Massaro and Egan, 1996). Using this paradigm, previous research has shown that the affective information transmitted by a voice (auditory) biases the affect perceived from faces (Massaro and Egan, 1996; de Gelder et al., 1999; de Gelder and Vroomen, 2000; Ethofer et al., 2006), from static affective body postures (Van den Stock et al., 2007), and from dynamic body movements that portray prototypical emotions (Van den Stock et al., 2008). Interestingly, Van den Stock et al. (2009) used two affective stimuli with low probability of co-occurrence in everyday life: affective whole body movements of everyday actions (drinking movements performed with six different emotional expressions; happy, sad, angry, disgust and neutral) and instrumental classical music. The emotional expression was achieved by asking the actors of the movements to imagine scenarios where they had felt the respective emotions and act out the drinking movement in that emotional state. The aim was to establish whether the cross-modal bias would persist. Results suggest that prior exposure to particular combinations of multi-modal affective stimuli does not seem to be necessary for the cross-modal bias to be evident. It is potentially an inherent capacity necessary for interpersonal communication (Vines et al., 2011), as it is not dependent on whether the two affective stimuli co-occur naturally.

Although the phenomenon of the cross-modal bias has been demonstrated numerous times, the affective mechanisms mediating it are still not fully understood but some sort of affective neuroentrainment seems to be involved: it is not just to rhythm that our brains resonate, but also to the affect expressed. Neural responses for cross-modal affective stimulation have been investigated using different neuroimaging techniques (EEG, fMRI and TMS). These studies show synchronized and enhanced neural activation in amygdala, striatal and frontal areas when the cross-modal affective information is presented bi-modally (visual and auditory), as compared to when the emotional information is presented only through one modality (visual or auditory) (Baumgartner et al., 2006a,b, 2007). Interestingly, activations in amygdala and striatum are commonly related to the experience of arousal at the psychological level, while frontal activations are related to the experience of different valence (Kreibig, 2010). It remains unclear what role different dimensions of stimulus valence and arousal play in mediating the cross-modal effect and to what extent cross-modal influences of valence and arousal impact not only our perceptions of affective stimuli (behavioral ratings), but also our psychophysiological responses to them (such as galvanic skin responses, GSR).

To address this issue in a more ecologically valid context, we examined the cross-modal bias in relation to affective responses to original dance video sequences taken from real performances (Christensen et al., 2014), with and without congruent classical music. This more ecological material provides an interesting phenomenological approach. While previous research established the cross-modal bias exclusively for the *perception* of emotion (i.e., what emotion a person *recognizes* in a display), how the *actual emotional experience* of an individual is affected by cross-modal affective information has not yet been investigated. It is an acknowledged difficulty in affective neuroscience to ensure that experimental participants are indeed genuinely experiencing emotions in response to experimental stimuli, and not merely recognizing them (Bastiaansen et al., 2009). Therefore, we choose real *music* and *dance* as stimulus materials. Music is an artful stimulus with an artistic and affective expression intrinsically linked to it (Blood and Zatorre, 2001; Salimpoor et al., 2009, 2013). The same is true for dance (Calvo-Merino et al., 2008; Jang and Pollick, 2011; Jola et al., 2011b; Grosbras et al., 2012; Christensen et al., under review). In addition, conversely to every-day expressive affective body movement, the normative affective connotations of dance movements cannot be known by individuals with neither visual nor motor experience with these movements (dance movements can be non-propositional, non-referential, non-every day movements; i.e., movements without any known affective cues that occur naturally in any interpersonal communication but are difficult to quantify, such as nodding or shaking the head, etc). Christensen and Calvo-Merino (2013) discussed available experimental works with regards to how such movement features contribute to the affective experience of an observer. Christensen et al. (2014) confirmed this at a behavioral level for the present stimuli set. We therefore hypothesize that any affective responses in the perceivers would likely be due to the genuine affect-inducing qualities of the movements. Thus, using a formalized type of dance such as ballet dance as experimental stimulus allows investigating the cross-modal bias while minimising top-down knowledge-based modulation of the affective experience.

Finally, most previous studies (exceptions: Van den Stock et al., 2009; Vines et al., 2011) have compared dynamic stimuli in the auditory modality (such as audio clips of voice recordings, animal sounds, music, etc) with static visual stimuli (mostly pictures of facial or bodily affective expressions). Therefore, by using music and dance, we follow the example of Vines et al. and Van der Stock et al., and maintain the dynamic property of the stimuli constant in both modalities. Considering the body of research available on the cross modal bias, we take it as a given that music presented alongside a dance is likely to “bias” the affective experience of that dance (= hypothesis 1). However, the role of arousal and valence in mediating the cross modal effect has not been empirically addressed. Therefore, in addition to the cross modal bias (= hypothesis 1), we hypothesized that the valence and arousal characteristics of the dance and its accompanying music would modulate participants’ responses (= hypothesis 2). Specifically, we predicted that particular pairings of valence and arousal characteristics in music and dance would result in higher affective responses than others. This idea is based on the observation that participants reliably relate particular movement features with sound configurations which they believe to match the movements (Sievers et al., 2013). It makes sense to suspect that this matching of sound and visual characteristics has something to do with the stimuli’s valence and arousal characteristics. Thus, for instance, at the end of *The Dying Swan* the decaying and lethargic final bars of the music somehow augment the slow “suffering” final movements of the choreography in the moment the swan is losing its death fight and succumbs on the floor. It is unlikely that without this particular pairing of music and movement the spectators would have the same high affective responses they have in this case. Therefore, we predict that low arousal dance and sad music; high arousal dance and happy music, on the other, will result in the strongest affective responses.

## Method

### Participants

Eighteen undergraduate psychology students (9 males) participated in exchange for course credits (age: *m* = 20.44; SD = 2.09). Participants completed an art experience questionnaire (Chatterjee et al., 2010). Scores between 0–14 designate artistically naïve individuals, while artistically experienced individuals have scores above 14. Participants’ mean score on the art experience questionnaire was 12.833 (SE = 2.293; SD = 9.727; range 1–32). Eleven participants had scores below 14 and seven participants had scores above 14. Thus, our sample was characterized by a relatively large range of more general art experience, but none of the participants had received professional dance training. Participants gave informed consent and the study was approved by the Research and Ethics Committee of the Department of Psychology of City University London.

For the purposes of a larger research project, we also asked participants to complete the Bermond-Vorst Alexithymia Questionnaire (BVAQ; Vorst and Bermond, 2001). The BVAQ includes five sub dimensions; *Emotionalizing* (awareness of own arousal level in response to emotional displays), *Fantasizing* (inclination to daydream, imagine), *Identifying* (ability to identify own arousal states), *Analyzing* (degree of introspection on own emotional reactions), and *Verbalizing* (ability to describe and express own emotional reactions). On the BVAQ participants had a mean score of 49.72 (SE = 2.077; SD = 8.810; range 31–67). However, results from this questionnaire will not be discussed in this paper.

### Materials

Forty-eight ballet dance video clips (24 expressing happiness, 24 sadness) were selected from an extensively normed ballet movement library (Christensen et al., 2014; *N* = 203). Positively and negatively valenced videos were chosen on the basis of (i) the emotion the clip was intended to portray (happy or sad); and (ii) ratings of valence (1 = very negative, 7 = very positive) and arousal (1 = very low, 7 = high) in the normative sample of the stimulus library. To permit examination of the independent influences of valence and arousal on the cross-modal bias, stimuli were carefully chosen to orthogonalize these dimensions. Thus, we ensured that half (i.e., 12) of each of the positively and negatively valenced dance stimuli comprised high arousal clips and the other low arousal clips. This classification was based on the previous normative study in which a total of 98 participants (dance-art naive individuals as well as dance professionals) were asked to provide valence, arousal and different movement-related ratings for the 203 ballet dance clips selected from various live performances (Christensen et al., 2014).

In addition to the dance stimuli, two music pieces served as experimental materials, which were selected based on a music stimulus validation procedure by Mitterschiffthaler et al. (2007). In a pilot experiment these authors asked 53 participants to rate 60 1-min pieces of 18th, 19th and 20th century classical music. Participants rated whether or not the piece would change their emotional state. If it did, their task was to indicate on a scale (from sad passing through neutral to happy) how sad, or happy it made them feel. Twenty clips were classified as sad, 20 as happy and 20 as neutral. For the present experiment two full versions of the music validated by this study were selected: happy music: *Blue Danube* by Johann Strauβ; sad music: *Adagio in G minor* by Tomaso Albioni. Both pieces were about 8 min long and were played to participants in relevant blocks of trials via headphones connected to an MP3 player (Ipod).

### Procedure

The experimental procedure consisted of three blocks of 48 trials each (i.e., 144 trials in total), with short breaks separating the blocks (the breaks were self-paced, lasting a couple of minutes). The 48 stimuli were the same in each of the three blocks, but displayed in random order. Throughout two of the blocks the happy and sad music was played whilst in the remaining block no music was played. This procedure was followed by Baumgartner et al. (2006a,b, 2007). The order of the three music conditions was counterbalanced across subjects and because the task was self-paced (i.e., participants had unlimited time to give their ratings), music was set to loop in case the block was longer than the musical piece (music was played 1–2 times each time, depending on the individual answer speed of each participant).

Dance videos were presented on a 15″ monitor powered by a PC desktop using E-prime software (version E-Studio, v. 2.0.8.90^1^), with participants sitting at a standard viewing distance of approximately 45 cm. Each video lasted 6 s and was presented in grey-scale on a black background in the center of the screen. Each dancer occupied approximately 5.5 cm on the screen (head to heal) and the dancers’ faces were blurred out to avoid participants extracting affective cues from facial expressions.

**Figure 1:**
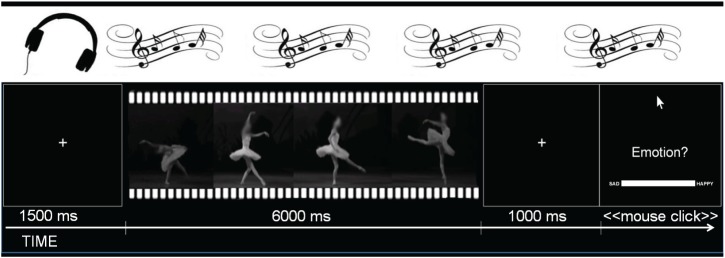
**Trial structure example**.

Before the experimental blocks, four practice trials were presented to familiarize participants with the procedure. Each block then began with a 10 s long interval of only music (silence for the control condition) during which participants were instructed to simply relax and wait for the task to begin. After these 10 s, the presentation of the ballet clips commenced automatically. After each clip, participants were asked to indicate how the clip made them *feel* on a sad-happy visual analogical scale (VAS). This scale was displayed horizontally in the lower half of the screen (21.4 cm long and 1.5 cm wide) with the labels “*Sad*” (left) and “*Happy*” (right) displayed on either side, while the prompt “*Emotion?*” was displayed in the center of the screen (see Figure 1) until the participant responded. Based on similar studies in our laboratory (Christensen et al., under review), the next trial started after 1.5 s of inter stimulus interval (ISI). For the statistical analyses, VAS ratings were quantified as ranging from 0 (Sad) to 100 (Happy). The numeric labels were not visible to the participants. The selection of this scale was based on Mitterschiffthaler et al. (2007). Instructions to the participants emphasized that they should ignore whatever they would be hearing in the headphones when providing their ratings and to focus their attention only on the emotion *the movement made them FEEL*.

### Autonomic arousal measurement

Galvanic Skin Responses were recorded throughout the experiment at a frequency of 1 kHz with an ADInstruments Power-Lab System (ML845) including a GSR (ML116) signal amplifier (ADInstruments, 1994–2004a,b). Stainless steel bipolar GSR electrodes (MLT116F) were attached to the medial phalanges of the index and ring fingers of the participant’s non-dominant hand using fitted velcro straps. A computer running LabChart 7 (v.7.3.1. 1994–2004^2^) recorded the GSR data and a parallel-to-serial port link with the stimulus presentation desktop ensured that the GSR data trace was demarcated with relevant stimulus events (i.e., video onsets). After visual inspection to screen for movement artefacts, and following standard procedures (Bradley et al., 2001), GSR responses were quantified by first subtracting the *maximum value* within the 6 s of the video stimulus from the GSR *value at the onset* of the stimulus and then applying a *log transformation* (log[GSR+1]) to normalize the distribution of the data. Due to technical difficulties, GSR was not available for one participant and for the last block (1 happy music block; 1 no music block) of two further participants. To minimize participant drop-out, the data values for the lost blocks of the latter two participants were replaced with the group means of the affected conditions though it is worth noting that exclusion of these participants from the analysis does not significantly alter the pattern of results reported below.

### Analyses

For the factors valence and arousal, VAS ratings and GSR data were analyzed using separate 3 × 2 repeated measures Analyses of Variances (ANOVAs) with Music condition (Happy-None-Sad) and Dance valence (Positive—Negative) as the within subjects factors. Following our predictions about the cross modal bias (hypothesis 1), and the fact that low arousal dance and sad music; high arousal dance and happy music, on the other, would elicit the strongest affective responses (hypothesis 2), interactions were followed up accordingly using planned comparisons (*t*-tests;), so that no corrections for multiple comparisons were required (Rothman, 1990; Saville, 1990; McDonald, 2009). As effect sizes we report Cohen’s *d* and eta squared (*η*^2^; Cohen, 1988).

## Results

### The role of valence

**Figure 2:**
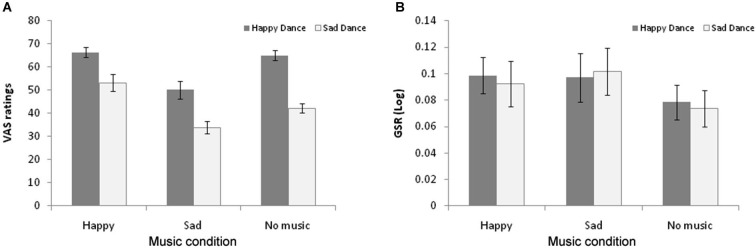
**(A)** Average VAS ratings of the Positive (grey) and Negative (white) dance stimuli as a function of Music condition. Lower numbers on the VAS scale represent more negative valence ratings. **(B)** Average peak Galvanic Skin Responses (log transformed) to Positive (grey) and Negative (white) dance stimuli as a function of Music condition. Error bars represent standard error mean (SEM).

To examine the role of valence in the cross-modal bias, VAS ratings and GSR data were analyzed using separate 3 × 2 repeated measures ANOVAs with Music condition (Happy-None-Sad) and Dance valence (Positive—Negative) as the within subjects factors. Shapiro-Wilk test of normality showed that the data were normally distributed. The analysis of VAS ratings revealed significant main effects for both Music condition (*F*_(2,34)_ = 14.510, *p* < 0.001, *η*^2^ = 0.460), and Dance valence (*F*_(1,17)_ = 66.185, *p* < 0.001, *η*^2^ = 0.796) as well as a significant interaction between the factors (*F*_(2,34)_ = 4.638, *p* < 0.05, *η*^2^ = 0.214). As shown in Figure 2A, the main effect of Music is explained by the fact that VAS ratings in the happy music condition (*M* = 59.75; SE = 2.45) were significantly more positive (*t*_(17)_ = 2.82; *p* < 0.05; Cohen’s *d* = 0.72), and ratings in the sad music condition (*M* = 41.34; SE = 2.92) significantly more negative (*t*_(17)_ = 3.575; *p* < 0.05; Cohen’s *d* = 1.23), than in the no music condition (*M* = 53.41; SE = 1.52), confirming the cross-modal bias. The main effect of dance valence furthermore validates the choice of our dance stimuli by confirming that happy movements received significantly more positive ratings (*M* = 64.24; SE = 2.17) than sad movements (*M* = 42.57; SE = 1.92; *t*_(17)_ = 8.138; *p* < 0.001; Cohen’s *d* = 2.49).

Finally, the source of the interaction was explored through paired *t*-tests that compared participants’ ratings between the music conditions for each type of dance emotion (happy vs. sad). This showed that sad dance stimuli were rated as significantly more positive (*t*_(17)_ = 3.32; *p* < 0.01; Cohen’s *d* = 0.93) in the happy music (incongruent) condition (*M* = 53.63; SE = 3.45) and as significantly more negative (*t*_(17)_ = 3.06; *p* < 0.01; Cohen’s *d* = 0.96) in the sad music (congruent) condition (*M* = 33.73; SE = 2.39) compared to the no music condition (*M* = 42.57; SE = 1.92). By contrast, happy dance stimuli, although rated as significantly more negative (*t*_(17)_ = 3.59; *p* < 0.01; Cohen’s *d* = 1.55) during the sad music (*M* = 48.95; SE = 3.85) compared to the no music condition (*M* = 64.24; SE = 2.17) were not rated more positively during the happy music condition (*M* = 65.91; SE = 1.97; *t*_(17)_ = 0.723; *ns*; Cohen’s *d* = 0.19). In other words, whereas the cross-modal bias was evident bi-directionally in the context of both happy and sad music for negative dance stimuli, it was only observed in the context of sad music for positive dance stimuli.

A similar analysis was performed on the GSR data. In Figure 2B, we can observe a tendency for lower GSR responses overall in the no music condition (*M* = 0.076; SE = 0.013) compared to the happy music (*M* = 0.096; SE = 0.014) and sad music (*M* = 0.099; SE = 0.018) conditions. A 3 (Music) × 2 (Dance Valence) ANOVA of the data revealed no main effects of music (*F*_(2,32)_ = 1.536; *ns*; *η*^2^ = 0.088) or dance valence (*F*_(1,16)_ = 0.680; *ns*; *η*^2^ = 0.011), nor an interaction between the factors (*F*_(2,32)_ = 0.310; *ns*; *η*^2^ = 0.019). Thus, neither the cross-modal bias nor the effect of dance valence that were observed in relation to participant’s VAS ratings were evident in relation to their physiological responses to the dance stimuli.

### The role of arousal

**Figure 3:**
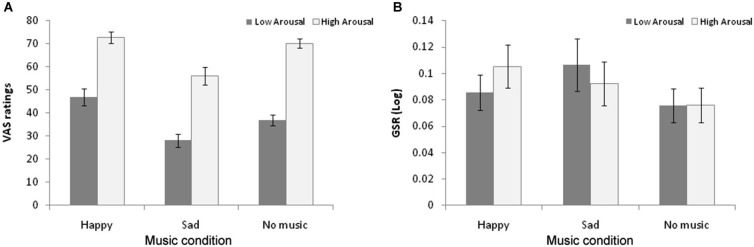
**(A)** Average VAS ratings of the Low-Arousal (grey) and High-Arousal (white) dance stimuli as a function of Music condition. Lower numbers on the VAS scale represent more negative valence ratings **(B)** Average peak Galvanic Skin Responses (log transformed) to High Arousal (white) and Low Arousal (grey) dance stimuli as a function of Music condition. Error bars represent standard error mean (SEM).

To examine the role of arousal in the cross-modal bias, we performed a similar analysis using high vs. low arousal as the within subjects variable of interest in relation to the dance stimuli. A 3 × 2 repeated measures ANOVA with Music condition (Happy-None-Sad) and Dance arousal (High-Low) was performed on participant’s VAS ratings. Shapiro-Wilk test of normality showed that the data were normally distributed. Results confirmed the main effect of music condition described above and also revealed a main effect of Dance arousal, with low-arousal videos (*M* = 37.71; SE = 1.77) receiving significantly lower VAS ratings (*t*_(17)_ = 9.77; *p* < 0.001; Cohen’s *d* = 3.44) than high-arousal videos (*M* = 65.28; SE = 8.49). The interaction between the factors was not significant (*F*_(2,34)_ = 2.599; *ns*; *η*^2^ = 0.133), indicating that the cross-modal bias operated to similar extents for both high-arousal and low-arousal dance stimuli (Figure 3A).

Finally, participants’ GSR data were also analyzed as a function of dance arousal. Shapiro-Wilk test of normality showed that the data were normally distributed. The 3 × 2 ANOVA revealed a significant 2-way interaction (*F*_(2,34)_ = 4.579; *p* < 0.05; *η*^2^ = 0.223) in the absence of any main effects. As Figure 3B suggests, and in line with a cross-modal bias, follow-up comparisons showed that GSR levels were significantly greater to low-arousal dance videos in the sad music (*M* = 0.107; SE = 0.020) compared to the no music (*M* = 0.076; SE = 0.013) condition (*t*_(16)_ = 2.269; *p* < 0.05; Cohen’s *d* = 0.45) whilst GSR levels to high-arousal dance clips were marginally greater in the happy music (*M* = 0.105; SE = 0.016) than the no music (*M* = 0.076; SE = 0.013) condition (*t*_(16)_ = 1.795; *p* = 0.077; Cohen’s *d* = 0.48). In other words, music congruent with the tone of arousal on display in dance movements (sad music for lethargic dance; happy music for energetic dance) augments participants’ physiological responses to the stimuli.

## Discussion and conclusion

The objective of the present experiment was to determine what role the emotional valence and arousal of a movement play in the cross-modal bias, both in terms of behavioral and physiological responses. Moreover, by employing video clips of real dance performances (Christensen et al., 2014), we extended previous research on the cross-modal bias in affect processing to more ecologically valid contexts. For empirical support for the increased ecological validity of experiments when using video clips to increase genuine affective experiences in affective neuroscience, see Carvalho et al. (2012); for discussions see Jola et al. (2011a); Christensen and Calvo-Merino (2013).

With regards to the role of *valence*, while confirming the cross-modal bias with these stimulus materials, we found that different movement* valences* mediated the cross-modal bias. In particular, an interaction indicated that participants’ subjective responses (VAS ratings) to the affective body movement were modulated by the affective valence of the music (sad or happy). The cross-modal bias was confirmed only in the case of *incongruent* cross-modal affective stimulation (i.e., happy movements rated as more sad with sad music, and sad movements rated as more happy with happy music). Conversely, when the cross-modal affective information was *congruent* (i.e., happy movements with happy music and sad movements with sad music), the valence of the movements made a difference: namely, sad music made the ratings to sad movements more sad. However, happy music did not make happy movements more happy. This suggests that in the context of happy movements the cross-modal bias was not fully evident. This is contrary to previous work which showed that affective ratings of both sadness and happiness in a photograph of a face was increased when congruent sad or happy classical music was played (Logeswaran and Bhattacharya, 2009), but in line with Jeong et al. (2011) who also reported the cross-modal bias clearly for the sad faces condition (sadness ratings to the faces more sad when paired with sad music), while it was marginally non-significant in the happy faces condition (*p* = 0.051). A reason for these divergent results could be the use of different methodologies. We used highly ecological and dynamic stimuli in both perceptual domains while the cited studies used laboratory-staged and static stimuli in the visual domain. Furthermore, it should be noted as a potential limitation that the music continued to play throughout participants giving their ratings. However, whilst this issue could account for the main effect of music (i.e., movements rated as more sad during sad music and happier during happy music), it cannot account for the observation of the cross-modal bias (that music influences ratings to sad and happy movements differently). Another possibility is that, perhaps the perception of happiness from genuinely happy movement is already at its maximum in the visual condition, thus, music does not add to this experience. In fact, Van den Stock et al. (2009) who also used dynamic stimuli in both modalities (body movement video clips and classical music)—reported a similar finding as ours. In particular, the proportion of “happy” responses was lowest when the music was sad (i.e., affective ratings tended to be sad not happy in the sad movement condition), while there were no significant differences for the other music conditions (happy music and no music; i.e., the addition of happy music to happy body movement did not make ratings predominantly happy as compared to a condition where the music was neutral). No effect of movement valence was found in the GSR of the participants. To explore this question further, a complementary future approach could combine heart rate (HR) data alongside GSR, as HR is known to provide a psychophysiological index of valence (Kreibig, 2010).

In terms of the two different levels of movement *arousal*, low arousal movements resulted in lower ratings on the VAS, and high arousal movements in higher ratings. No interaction was found which suggests that the cross-modal bias occurs equally for low and high arousal movements. Conversely, movement arousal made a difference for the participants’ physiological response. In accordance with our hypotheses, GSR were higher in the case of low arousal movements paired with sad music (as compared to happy music), and in the case of high arousal movements paired with happy music (as compared to sad music). Thus, when the music dynamics were in tone with the arousal dynamics of the movements, people had higher physiological arousal. It could seem odd that physiological arousal should be heightened during both positive and negative stimuli (happy-sad) with different arousal dynamics (low-high). However, such effect has often been reported (i.e., comparably high responses for happy, sad, angry, fearful, anxious, etc.), both at physiological level (heart rate, GSR, breathing rate, etc.) and at neural level (evidenced by activation of limbic structures), both for non-art stimuli (Bradley and Lang, 2000), and musical pieces (VanderArk and Ely, 1992, 1993; Khalfa et al., 2005; Pallesen et al., 2005; Green et al., 2008). Our observations imply that in a multi-modal context the mere matching of valence in movement (visual) and music (auditory) is not enough to produce an effect on physiological arousal. Rather, also movement and music dynamics have to be matched in a particular way for the effect to occur. In musical compositions tempo changes are a common and universal way to induce affect in observers (Holbrook and Anand, 1990). Sad mood is induced with lethargic and low arousal music, while happy mood is induced with energetic and high arousal music. It appears that when music and movement are presented together, it is the particular combination of features of the music and movement—their respective *dynamics*—that drives the psychophysiological responses of the perceivers, not the mere matching of valence (e.g., sad music with sad movement)—nor the mere matching of arousal (e.g., low arousal music with low arousal movement), but their interaction (e.g., sad arousal music with sad movement—not high arousal music with sad movement). This is in accordance with previous behavioral results. Visual and auditory stimuli of a particular valence and arousal level have been shown to share a common structure (Krumhansl and Schenck, 1997; Eitan and Granot, 2006), an effect that holds even cross-culturally. When asked to express emotion in music and movement, two culturally diverse groups of participants (US students and inhabitants of a remote Cambodian village) choose the same music and movement structure (rate, jitter (regularity of rate), direction, step size, and dissonance/visual spikiness) for each of the emotions they were asked to express (Sievers et al., 2013).

In other words, movement valence matters in the context of the cross-modal bias in participants’ subjective responses: the full cross-modal bias (both in the congruent and incongruent condition) in subjective responses only occurs with negatively valenced movements. Besides, movement valence does not impact peoples’ GSR. Conversely, movement arousal does not matter in the context of the subjective responses; the cross-modal bias is evident equally for low and high arousal movements. However, there is a clear cross-modal bias in participants’ physiological response (GSR). Galvanic Skin Responses is higher in the case where movement arousal is congruent with the musical tone. This suggests that the addition of music in a sad ballet piece like *Swan lake* (often slow and lethargic) will not only change the spectators affective perception of the affective expressive quality of the movements, but also their physiological responses to what they see. The same is the case when the arousal tone of a piece is high and happy music is played at the same time.

Interestingly, these congruency effects have not yet been reported at the neural level in the same manner. What has been shown is, for example, that the combination of visual-auditory emotional information evokes stronger responses in emotional areas linked to automatic emotion or feelings (amygdala, hippocampus, parahippocampus, insula, striatum), while the unimodal presentation of emotional stimuli activates brain regions more associated with the cognitive part of the emotional process (prefrontal cortices) (Baumgartner et al., 2006b). However, while both the automatic emotional and cognitive emotional areas show sensitivity to the combination of multisensory emotional information, it has not yet been confirmed whether any of these areas show sensitivity to the specific congruency (of valence and arousal) between the visual and auditory stimuli. The main effect of an enhanced affective response to multimodal emotional information—as compared to unimodal information—has been replicated by at least two additional studies using different neuroimaging techniques such as EEG (Baumgartner et al., 2006a), and TMS (Baumgartner et al., 2007). Thus, this effect can be assumed as robust. It remains to be established by future studies combining the measurement of behavioral, autonomic and neural responses simultaneously, how particularly the cross modal integration is mediated by congruence of the affective information in terms of stimulus valence and arousal.

Physiological theories suggest that the physiological arousal response (e.g., GSR) is the result of the perception of a stimulus that for some reason is significant to the organism (James, 1894; Schachter and Singer, 1962; LeDoux, 2003; Vuilleumier, 2005; Laird and Lacasse, 2014), thus providing support for neuroentrainment at the affective level. This physiological arousal response is often described as part of a threat-detection mechanism, important for survival in the wild because it alerts the organism and prompts appropriate behavioral responses (LeDoux, 2003, 2012). However, it can also be seen simply as a mechanism by which it is evidenced whether or not an individual engages with a stimulus because it is, for some reason, emotionally relevant to them (Shapiro et al., 1997), or more particularly, because it is perceived as expressive of an emotional state. In this context, it has been described previously that the valence and arousal interact in the elicitation of physiological arousal and that this arousal designates an enhanced engagement with such stimuli (e.g., shown by heightened performance in an attentional task in conditions of pairing low arousal-sad and high arousal-happy, Jefferies et al., 2008). Our results, in turn, illustrate that there are particular pairings of music and dance dynamics that result in a stronger affective engagement of the spectators than others.

In dance, theatre and cinema alike, directors have used music ever since to great effect in order to regulate the audience’s emotions. Thus, the finding of an interaction between valence and arousal in producing heightened psychophysiological responses in the perceiver is especially important in the context of in relation to the dialogue between arts and science. The present data suggest that the affective experience derived from affective body movement is best potentiated if movement arousal and music tone are in accordance (i.e., low arousal movements with sad music and high arousal movements with happy music produce the strongest physiological responses). Thus, when *Odette*—the swan princess of *Swan Lake*—dances her prayer to the hunter-prince, begging him not to shoot her sisters, her anxious sadness over the potential imminent catastrophe is evident in every single movement of her body. However, from the results presented here, we may assume that the reason why the spectators feel Odette’s suffocating desperation in their own bodies along with their own palms sweating (e.g., measured with GSR) is down to the conjunction of her longing and contained movements with the low arousal and sad tones of Tschaikovski’s oboe solo in that moment of the choreography (Petipa et al., 1895).

Knowing whether an audience engages with (and has psychophysiologically measurable responses to) an artwork may be of great interest in the art context (Latulipe et al., 2011). Sure, choreographers and artists in general will not be primarily concerned with audience responses *per se*, but will use their own phenomenological experience as an indicator for the “goodness” of an artwork. It is also common to hear that artist don’t care what others think and feel about their art. They just “do” it. However, scientific evidence such as the one presented here may be helpful to other people invariably linked to the art world (such as agents or producers) to avoid dressing the Emperor’s* New Clothes* (i.e., supporting hyped “art” that does not genuinely engage spectators)^3^. Also choreographers and directors still in training may be interested in knowing “tools” to reliably engage their future audiences (such as successfully pairing music and dance dynamics). One may argue that enhanced physiological arousal can also be the signature of a dislike response. While this is true, such a response still illustrates an engagement with the piece. Take for instance Stravinsky’s dissonant Music and Massine’s Rite of Spring choreography (Nijinsky et al., 1913/1920). It has been shown to clearly induce physiological arousal (Taylor, 1973). Yet, few pieces in the history of dance have elicited so much controversy (even outrage on its première!). But, this is still engagement. The piece received attention like few others, a phenomenon that is good for any artist, director, and producer. Economists will agree on this.

Hence, we suggest that scientific evidence such as that presented here can in any case inform the dance art community about audience engagement with their art. Previous dance neuroesthetics research has shown that energetic dance movements such as jumps or very difficult leg stretches are liked more and result in increased neural responses in spectators (Calvo-Merino et al., 2008; Cross et al., 2011). This research coined the concept of *choreographing for the esthetic brain* and suggested that the knowledge about these enhanced brain responses to particular kinematic properties of movements could be taken into account when developing new dance phrases (Calvo-Merino et al., 2008). Because dance is often a multisensory experience, our results, and others along the same lines (e.g., such that has demonstrated that powerful music elicits strong psychophysiological responses in perceivers; Harrer and Harrer, 1977; Rickard, 2004) could be taken into consideration to understand audience responses to a performance. This work also suggests an area where neuroentraintment research needs to pay more attention: where interaction matters, so that instead of just considering the adaptation of a system to an environmental stimulus, neuroentrainment is viewed as a reciprocal process of mutual adjustment, both at the sensorimotor and affective levels (Gomila, 2009). Future science and art collaborations could further explore the idea of a holistic and multisensory choreographic process, where the audience’s behavioral, psychological and neural responses inform the creative artistic process.

In conclusion, we have shown how the effect of music on the perception of a dance should never be underestimated. Music and dance valence and arousal dynamics interact to create the strongest affective experience from a dance piece. We reproduced the cross-modal bias with this type of non-every day affective body movement and instrumental classical music. This is another data point to suggest that the perception of affective cues is not depended on familiarity or particular experience with the affective cue. The cross-modal integration of affective information seems an inherent capacity of the human affect perception system, dependent more on the cue (its valence and arousal dynamics) than on prior experience with the cue.

## Conflict of interest statement

The authors declare that the research was conducted in the absence of any commercial or financial relationships that could be construed as a potential conflict of interest.

## Footnotes

^1^www.pstnet.com

^2^www.adinstruments.com

^3^Critics may see a danger in this in that producers will base decisions to fund and exhibit artists only based on such “objective” feedback. But then, If an art work does not engage its audience, it just isn’t the right art work for that audience, is it? That may be hurtful, but it doesn’t necessarily mean the art work is not “good”. It may simply not be it’s time yet. Many artists throughout history were also not appreciated by their contemporary audiences. Still, they never gave up their art and they left humanity a heritage that today no one denies.

## References

[B1] ADInstruments (1994–2004a). LabChart 7 v7.3.1. ADInstruments Pty Ltd. Psychology Software Tools (1996–2010). E-Prime 2.0 (2.0.8.90).

[B2] ADInstruments (1994–2004b). PowerLab Data acquisition unit (ML845) with GSR and Bioelectrical signal amplifiers and cables (ML116, MLT116F, ML408, MLA2540; MLA2505; MLA1010B). ADInstruments Pty Ltd.

[B7] Bastiaansen J. A. Thioux M. Keysers C. (2009). Evidence for mirror systems in emotions. Philos. Trans. R. Soc. Lond. B Biol. Sci. 364, 2391–2404 10.1098/rstb.2009.0058 19620110 PMC2865077

[B8] Baumgartner T. Esslen M. Jäncke L. (2006a). From emotion perception to emotion experience: emotions evoked by pictures and classical music. Int. J. Psychophysiol. 60, 34–43 10.1016/j.ijpsycho.2005.04.007 15993964

[B9] Baumgartner T. Lutz K. Schmidt C. F. Jäncke L. (2006b). The emotional power of music: how music enhanced the feeling of affective pictures. Brain Res. 1075, 151–164 10.1016/j.brainres.2005.12.065 16458860

[B10] Baumgartner T. Willi M. Jäncke L. (2007). Modulation of corticospinal activity by strong emotions evoked by pictures and classical music: a transcranial magnetic stimulation study. Neuroreport 18, 261–265 10.1097/wnr.0b013e328012272e 17314668

[B11] Béjart M. Ravel M. (1961). Boléro. Belgium, Bruxelles

[B12] Blood A. J. Zatorre R. J. (2001). Intensely pleasurable responses to music correlate with activity in brain regions implicated in reward and emotion. Proc. Natl. Acad. Sci. U S A 98, 11818–11823 10.1073/pnas.191355898 11573015 PMC58814

[B13] Bradley M. M. Codispoti M. Cuthbert B. Lang P. J. (2001). Emotion and motivation I: defensive and appetitive reactions in picture processing. Emotion 3, 276–298 10.1037//1528-3542.1.3.276 12934687

[B14] Bradley M. M. Lang P. J. (2000). “Measuring emotion: behavior, feeling and physiology,” in Cognitive Neuroscience of Emotion, eds Lane R. D. Nadel L. Ahern G. (New York: Oxford University Press), 242–276

[B15] Calvo-Merino B. Jola C. Glaser D. E. Haggard P. (2008). Towards a sensorimotor aesthetics of performing art. Conscious. Cogn. 17, 911–922 10.1016/j.concog.2007.11.003 18207423

[B16] Carvalho S. Leite J. Galdo-Álvarez S. Gonçalves O. F. (2012). The Emotional Movie Database (EMDB): a self-report and psychophysiological study. Appl. Psychophysiol. Biofeedback 37, 279–294 10.1007/s10484-012-9201-6 22767079

[B17] Chatterjee A. Widick P. Sternschein R. Smith Ii W. B. Bromberger B. (2010). The assessment of art attributes. Empir. Stud. Arts 28, 207–222 10.2190/em.28.2.f

[B18] Christensen J. F. Calvo-Merino B. (2013). Dance as a subject for empirical aesthetics. Psychol. Aesthet. Creat. Arts 7, 76–88 10.1037/a0031827

[B20] Christensen J. F. Nadal M. Cela-Conde C. J. Gomila A. (2014). A norming study and library of 203 dance movements. Perception 43, 178–206 10.1068/p7581 24919352

[B21] Cohen J. (1988). Statistical Power Analysis for the Behavioral Sciences. Hillsdale, NJ: Lawrence Erlbaum Associates Inc

[B22] Cohen A. J. (2010). “Music as a source of emotion in film,” in Handbook of Music and Emotion, eds Juslin P. Sloboda J. (Oxford: Oxford University Press), 879–908

[B23] Cross E. S. Kirsch L. Ticini L. F. Schütz-Bosbach S. (2011). The impact of aesthetic evaluation and physical ability on dance perception. Front. Hum. Neurosci. 5:102 10.3389/fnhum.2011.00102 21960969 PMC3177045

[B24] de Gelder B. Böcker K. B. Tuomainen J. Hensen M. Vroomen J. (1999). The combined perception of emotion from voice and face: early interaction revealed by human electric brain responses. Neurosci. Lett. 260, 133–136 10.1016/s0304-3940(98)00963-x 10025717

[B25] de Gelder B. Vroomen J. (2000). The perception of emotions by ear and by eye. Cogn. Emot. 14, 289–311 10.1080/026999300378824

[B26] Eitan Z. Granot R. Y. (2006). How music moves: musical parameters and listeners’ images of motion. Music Percept. 23, 221–247 10.1525/mp.2006.23.3.221

[B27] Ethofer T. Anders S. Erb M. Droll C. Royen L. Saur R. (2006). Impact of voice on emotional judgment of faces: an event-related fMRI study. Hum. Brain Mapp. 27, 707–714 10.1002/hbm.20212 16411179 PMC6871326

[B28] Gomila A. (2009). “Musical expression and the second person perspective,” in Expression in the Performing Arts, eds Alvarez I. Pérez F. Pérez H. (Cambridge: Cambridge Scholars Publishing), 66–85

[B29] Green A. C. Bærentsen K. B. Stødkilde-Jørgensen H. Wallentin M. Roepstorff A. Vuust P. (2008). Music in minor activates limbic structures: a relationship with dissonance? Neuroreport 19, 711–715 10.1097/WNR.0b013e3282fd0dd8 18418244

[B30] Grosbras M.-H. Tan H. Pollick F. (2012). Dance and emotion in posterior parietal cortex: a low-frequency rTMS study. Brain Stimul. 5, 130–136 10.1016/j.brs.2012.03.013 22494828

[B31] Harrer G. Harrer H. (1977). “Music, emotion and autonomic function,” in Music and the Brain: Studies in the Neurology of Music, eds Critchley M. Henson R. A. (London: William Heinemann), 202–216

[B32] Holbrook M. B. Anand P. (1990). Effects of tempo and situational arousal on the listener’s perceptual and affective responses to music. Psychol. Music 18, 150–162 10.1177/0305735690182004

[B33] James W. (1894). Discussion: the physical basis of emotion. Psychol. Rev. 1, 516–529 10.1037/h0065078 8022955

[B34] Jang S. H. Pollick F. E. (2011). Experience influences brain mechanisms of watching dance. Dance Res. J. 29, 352–377 10.3366/drs.2011.0024

[B35] Jefferies L. N. Smilek D. Eich E. Enns J. T. (2008). Emotional valence and arousal interact in attentional control. Psychol. Sci. 19, 290–295 10.1111/j.1467-9280.2008.02082.x 18315803

[B36] Jeong J. W. Diwadkar V. A. Chugani C. D. Sinsoongsud P. Muzik O. Behen M. E. (2011). Congruence of happy and sad emotion in music and faces modifies cortical audiovisual activation. Neuroimage 54, 2973–2982 10.1016/j.neuroimage.2010.11.017 21073970

[B37] Jola C. Ehrenberg S. Reynolds D. (2011a). The experience of watching dance: phenomenological-neuroscience duets. Phenomenol. Cogn. Sci. 11, 17–37 10.1007/s11097-010-9191-x

[B38] Jola C. Pollick F. E. Grosbras M.-H. (2011b). Arousal decrease in sleeping beauty: audiences’ neurophysiological correlates to watching a narrative dance performance of two-and-a-half hours. Dance Res. J. 29, 378–403 10.3366/drs.2011.0025

[B39] Khalfa S. Schon D. Anton J. L. Liégeois-Chauvel C. (2005). Brain regions involved in the recognition of happiness and sadness in music. Neuroreport 16, 1981–1984 10.1097/00001756-200512190-00002 16317338

[B40] Kreibig S. D. (2010). Autonomic nervous system activity in emotion: a review. Biol. Psychol. 84, 394–421 10.1016/j.biopsycho.2010.03.010 20371374

[B41] Krumhansl C. L. Schenck D. L. (1997). Can dance reflect the structural and expressive qualities of music? A perceptual experiment on Balanchine’s choreography of Mozart’s Divertimento No. 15. Music. Sci. 1, 63–85

[B42] Laird J. D. Lacasse K. (2014). Bodily influences on emotional feelings: accumulating evidence and extensions of William James’s theory of emotion. Emot. Rev. 6, 27–34 10.1177/1754073913494899

[B43] Latulipe C. Carroll E. A. Lottridge D. (2011). “Love, hate, arousal and engagement: exploring audience responses to performing arts,” in Proceedings of ACM CHI 2011 (Vancouver, BC, Canada: ACM), 1845–1854

[B44] LeDoux J. E. (2003). The emotional brain, fear and the amygdala. Cell. Mol. Neurobiol. 23, 727–738 10.1023/A:1025048802629 14514027 PMC11530156

[B45] LeDoux J. E. (2012). Rethinking the emotional brain. Neuron 73, 653–676 10.1016/j.neuron.2012.02.004 22365542 PMC3625946

[B46] Logeswaran N. Bhattacharya J. (2009). Crossmodal transfer of emotion by music. Neurosci. Lett. 455, 129–133 10.1016/j.neulet.2009.03.044 19368861

[B47] Massaro D. W. Egan P. B. (1996). Perceiving affect from the voice and the face. Psychon. Bull. Rev. 3, 215–221 10.3758/BF03212421 24213870

[B48] McDonald J. H. (2009). Handbook of Biological Statistics. 2nd Edn. Baltimore, Maryland: Sparky House Publishing

[B49] Mitterschiffthaler M. T. Fu C. H. Y. Dalton J. A. Andrew C. M. Williams S. C. R. (2007). A functional MRI study of happy and sad affective states induced by classical music. Hum. Brain Mapp. 28, 1150–1162 10.1002/hbm.20337 17290372 PMC6871455

[B50] Nijinsky V. Massine L. Stravinsky I. (1913/1920). The Rite Of Spring. Paris: Theatre des Champs-Elysees

[B51] Pallesen K. J. Brattico E. Bailey C. Korvenoja A. Koivisto J. Gjedde A. (2005). Emotion processing of major, minor, and dissonant chords: a functional magnetic resonance imaging study. Ann. N Y Acad. Sci. 1060, 450–453 10.1196/annals.1360.047 16597801

[B53] Perrot J. Coralli J. Adam A. (1841). Gisèlle. Paris, France

[B54] Petipa M. Iwanow L. Tschaikowski P. I. (1895). Swan Lake. St. Petersburg: Mariinski Theatre

[B55] Rickard N. S. (2004). Intense emotional responses to music: a test of the physiological arousal hypothesis. Psychol. Music 32, 371–388 10.1177/0305735604046096

[B56] Rothman K. J. (1990). No adjustments are needed for multiple comparisons. Epidemiology 1, 43–46 10.1097/00001648-199001000-00010 2081237

[B57] Salimpoor V. N. Benovoy M. Longo G. Cooperstock J. R. Zatorre R. J. (2009). The rewarding aspects of music listening are related to degree of emotional arousal. PLoS One 4:e7487 10.1371/journal.pone.0007487 19834599 PMC2759002

[B58] Salimpoor V. van den Bosch I. Kovacevic N. McIntosh A. R. Dagher A. Zatorre R. J. (2013). Interactions between the nucleus accumbens and auditory cortices predict music reward value. Science 340, 216–219 10.1126/science.1231059 23580531

[B59] Saville D. J. (1990). Multiple comparison procedures: the practical solution. Am. Stat. 44, 174–180 10.2307/2684163

[B60] Schachter S. Singer J. E. (1962). Cognitive, social and physiological determinants of emotional state. Psychol. Rev. 69, 379–399 10.1037/h0046234 14497895

[B61] Schneider F. Gur R. C. Gur R. E. Muenz L. R. (1994). Standardized mood induction with happy and sad facial expressions. Psychiatry Res. 51, 19–31 10.1016/0165-1781(94)90044-2 8197269

[B62] Shapiro K. L. Caldwell J. Sorensen R. E. (1997). Personal names and the attentional blink: a “visual” cocktail party effect. J. Exp. Psychol. Hum. Percept. Perform. 23, 504–514 10.1037//0096-1523.23.2.504 9104007

[B63] Sievers B. Polansky L. Casey M. Wheatley T. (2013). Music and movement share a dynamic structure that supports universal expressions of emotion. Proc. Natl. Acad. Sci. U S A 110, 70–75 10.1073/pnas.1209023110 23248314 PMC3538264

[B64] Stein B. E. London N. Wilkinson L. K. Price D. D. (1996). Enhancement of perceived visual intensity by auditory stimuli: a psychophysical analysis. J. Cogn. Neurosci. 8, 497–506 10.1162/jocn.1996.8.6.497 23961981

[B65] Taylor D. B. (1973). Subject responses to precategorized stimulative and sedative music. J. Music Ther. 10, 86–94 10.1093/jmt/10.2.86

[B66] Van den Stock J. Grèzes J. de Gelder B. (2008). Human and animal sounds influence recognition of body language. Brain Res. 1242, 185–190 10.1016/j.brainres.2008.05.040 18617158

[B67] Van den Stock J. B. Peretz I. Grèzes J. de Gelder B. (2009). Instrumental music influences recognition of emotional body language. Brain Topogr. 21, 216–220 10.1007/s10548-009-0099-0 19588251 PMC2707860

[B68] Van den Stock J. Righart R. de Gelder B. (2007). Body expressions influence recognition of emotions in the face and voice. Emotion 7, 487–494 10.1037/1528-3542.7.3.487 17683205

[B69] VanderArk S. D. Ely D. (1992). Biochemical and galvanic skin responses to music stimuli by college students in biology and music. Percept. Mot. Skills 74, 1079–1090 10.2466/pms.1992.74.3c.1079 1501973

[B70] VanderArk S. D. Ely D. (1993). Cortisol, biochemical and galvanic skin responses to music stimuli of different preference values by college students in biology and music. Percept. Mot. Skills 77, 227–234 10.2466/pms.1993.77.1.227 8367245

[B71] Vines B. W. Krumhansl C. L. Wanderley M. M. Dalca I. M. Levitin D. J. (2011). Music to my eyes: cross-modal interactions in the perception of emotions in musical performance. Cognition 118, 157–170 10.1016/j.cognition.2010.11.010 21146164

[B74] Vorst H. C. M. Bermond B. (2001). Validity and reliability of the Bermond-Vorst Alexithmia Questionnaire. Pers. Individ. Dif. 30, 413–434 10.1016/s0191-8869(00)00033-7

[B72] Vroomen J. de Gelder B. (2000). Sound enhanced visual perception: cross-modal effects of auditory organization on vision. J. Exp. Psychol. Hum. Percept. Perform. 26, 1583–1590 10.1037//0096-1523.26.5.1583 11039486

[B73] Vuilleumier P. (2005). How brains beware: neural mechanisms of emotional attention. Trends Cogn. Sci. 9, 585–594 10.1016/j.tics.2005.10.011 16289871

